# Supplementary material for: Evaluation of Anti-Oxinflammatory and ACE-Inhibitory Properties of Protein Hydrolysates Obtained from Edible Non-Mulberry Silkworm Pupae (Antheraea assama and Philosomia ricinii)
Source: Nutrients. 2023 Feb 19;15(4):1035. doi: 10.3390/nu15041035 (PMC9964498; doi:10.3390/nu15041035)
Supplement: Supplementary file 1 [file nutrients-15-01035-s001.zip › nutrients-2207221-supplementary.pdf]

Supplementary Data

Novel peptides purification from edible pupae hydrolysate of *Antheraea assama* "Muga" and *Philosomia ricinii* "Eri": angiotensin-I converting enzyme (ACE) inhibition and anti-oxinflammatory potency in LPS stimulated HUVECs cell line

Evaluation of antioxinflammatory and ACE-inhibitory properties of protein hydrolysates obtained from non-mulberry silkworm pupae *(Antheraea assama* and *Philosomia ricinii)*

Preeti Sarkar[a], Alessandra Pecorelli[b,c], Brittany Woodby[c], Erika Pambianchi[c], Francesca Ferrara[c,d], Raj Kumar Duary [a,g]*, Giuseppe Valacchi[b,c,e,] *,

[a]Department of Food Engineering and Technology, School of Engineering, Tezpur University, Assam, India.
[b]Department. of Environmental Sciences and Prevention, University of Ferrara, Ferrara, Italy.
[c]Plants for Human Health Institute, NC Research Campus, NC State University, NC, USA.
[d]Department of Neuroscience and Rehabilitation, University of Ferrara, Ferrara, Italy.
[e]Department of Food and Nutrition, Kyung Hee University, Seoul, South Korea.
[g]Department of Dairy Science & Food Technology, Institute of Agricultural Sciences, Banaras Hindu University (BHU), Varanasi, U.P., India.

***Corresponding authors:** Professor Giuseppe Valacchi, E-mail address: gvalacc@ncsu.edu, Professor Raj Kumar Duary, E-mail address: rkduary@bhu.ac.in,

Figure. S1: Correlation and regression analysis of degree of hydrolysis (%) with ACE inhibition activities (%) obtained with Muga pupae derived hydrolysates (A) and Eri pupae derived hydrolysates (B).

A

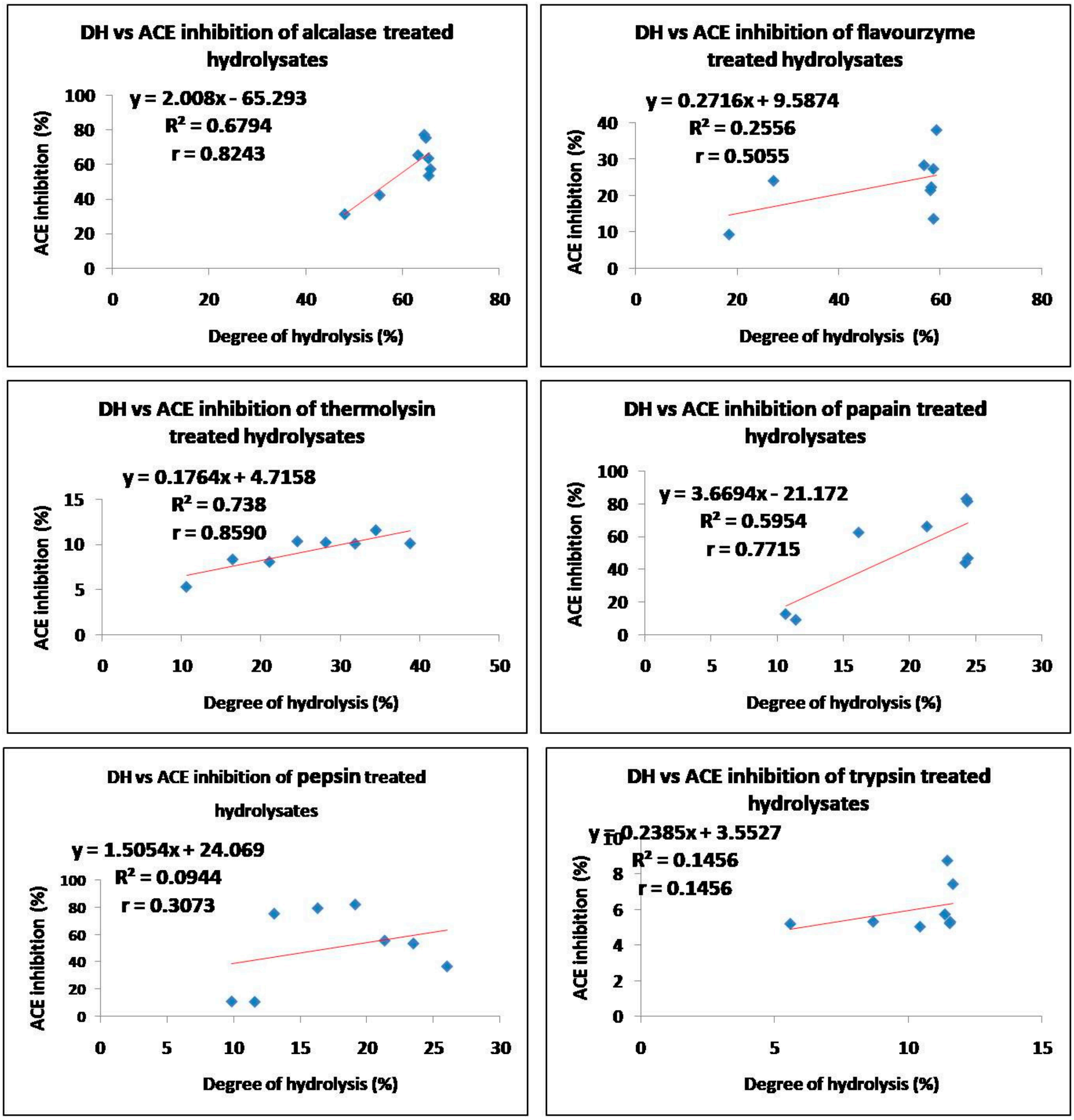

B

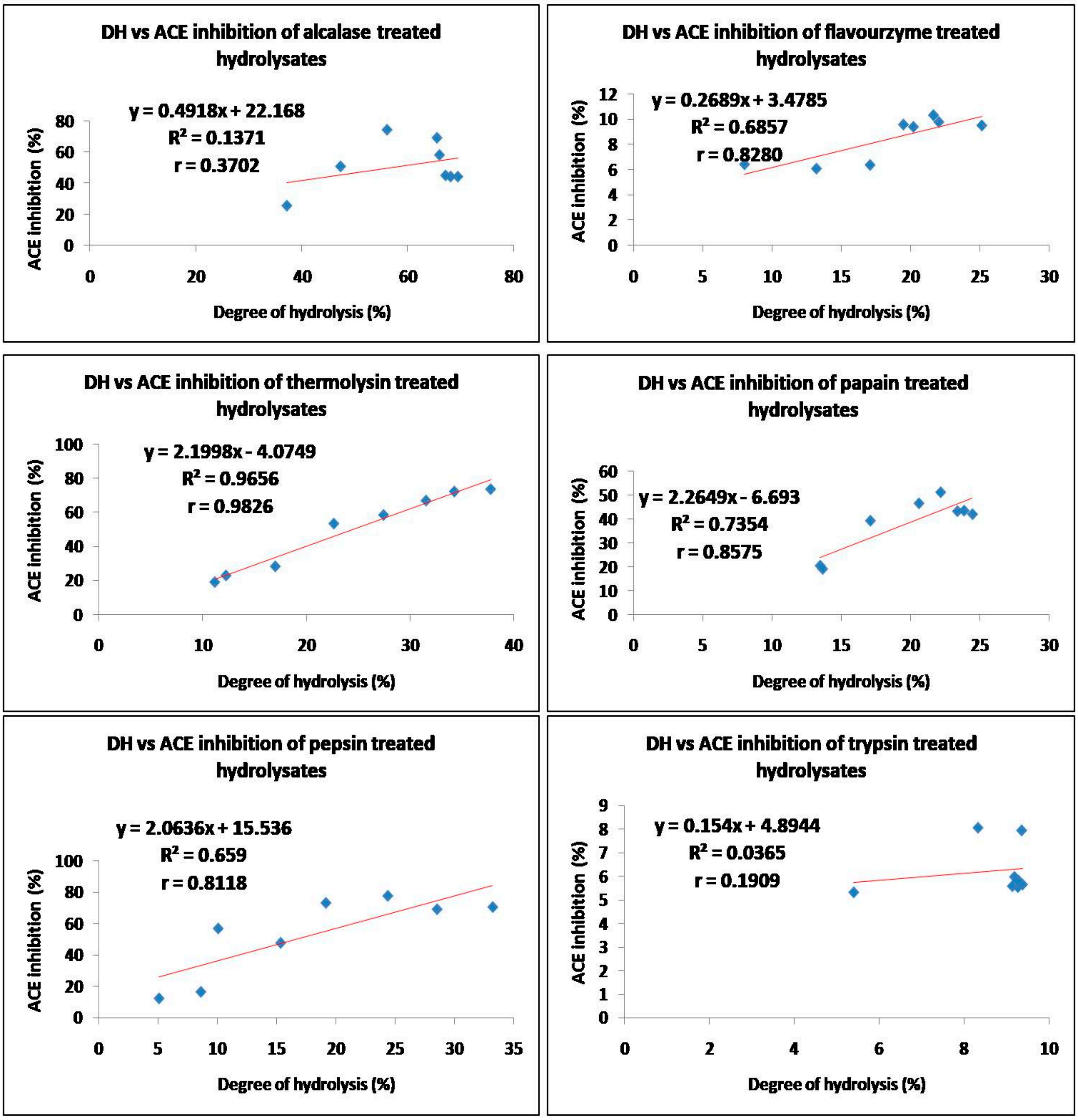
DH vs ACE inhibition of alcalase treated hydrolysates
y = 0.4918x + 22.168
R² = 0.1371
r = 0.3702
ACE inhibition (%)
Degree of hydrolysis (%)
DH vs ACE inhibition of flavourzyme treated hydrolysates
y = 0.2689x + 3.4785
R² = 0.6857
r = 0.8280
ACE inhibition (%)
Degree of hydrolysis (%)
DH vs ACE inhibition of thermolysin treated hydrolysates
y = 2.1998x - 4.0749
R² = 0.9656
r = 0.9826
ACE inhibition (%)
Degree of hydrolysis (%)
DH vs ACE inhibition of papain treated hydrolysates
y = 2.2649x - 6.693
R² = 0.7354
r = 0.8575
ACE inhibition (%)
Degree of hydrolysis (%)
DH vs ACE inhibition of pepsin treated hydrolysates
y = 2.0636x + 15.536
R² = 0.659
r = 0.8118
ACE inhibition (%)
Degree of hydrolysis (%)
DH vs ACE inhibition of trypsin treated hydrolysates
y = 0.154x + 4.8944
R² = 0.0365
r = 0.1909
ACE inhibition (%)
Degree of hydrolysis (%)
